# Five consecutive epidemiological waves of COVID-19: a population-based cross-sectional study on characteristics, policies, and health outcome

**DOI:** 10.1186/s12879-022-07909-y

**Published:** 2022-12-05

**Authors:** Rozhin Amin, Mohammad-Reza Sohrabi, Ali-Reza Zali, Khatereh Hannani

**Affiliations:** 1 Community Medicine Department, School of Medicine, Shahid Beheshti University of Medical Sciences, Tehran, 19839-63113 Iran; 2 Social Determinants of Health Research Centre, Shahid Beheshti University of Medical Sciences, Tehran, 19839-63113 Iran; 3 Functional Neurosurgery Research Centre, Shahid Beheshti University of Medical Sciences, Tehran, 19839-63113 Iran; 4 Statistics and Information Technology Management, Shahid Beheshti University of Medical Sciences, Tehran, 19839-63113 Iran

**Keywords:** COVID-19, Epidemics, Epidemiology, Iran, Pandemics, Population characteristics

## Abstract

**Background:**

This study was conducted with the intension of providing a more detailed view about the dynamics of COVID-19 pandemic. To this aim, characteristics, implemented public health measures, and health outcome of COVID-19 patients during five consecutive waves of the disease were assessed.

**Methods:**

This study was a population-based cross-sectional analysis of data on adult patients who were diagnosed with COVID-19 during five waves of the disease in Iran. Chi-squared test, One-way ANOVA, and Logistic Regression analysis were applied. A detailed literature review on implemented public health policies was performed by studying published documents and official websites responsible for conveying information about COVID-19.

**Results:**

Data on 328,410 adult patients was analyzed. Main findings indicated that the probability of dying with COVID-19 has increased as the pandemic wore on, showing its highest odd during the third wave (odds ratio: 1.34, CI: 1.283–1.395) and has gradually decreased during the next two waves. The same pattern was observed in the proportion of patients requiring ICU admission (P < 0.001). First wave presented mainly with respiratory symptoms, gastrointestinal complaints were added during the second wave, neurological manifestations with peripheral involvement replaced the gastrointestinal complaints during the third wave, and central nervous system manifestations were added during the fourth and fifth waves. A significant difference in mean age of patients was revealed between the five waves (P < 0.001). Moreover, results showed a significant difference between men and women infected with COVID-19, with men having higher rates of the disease at the beginning. However, as the pandemic progressed the proportion of women gradually increased, and ultimately more women were diagnosed with COVID-19 during the fifth wave. Our observations pointed to the probability that complete lockdowns were the key measures that helped to mitigate the virus spread during the first twenty months of the pandemic in the country.

**Conclusion:**

A changing pattern in demographic characteristics, clinical manifestations, and severity of the disease has been revealed as the pandemic unfolded. Reviewing COVID-19-related public health interventions highlighted the importance of immunization and early implementation of restrictive measures as effective strategies for reducing the acute burden of the disease.

## Background

The ongoing pandemic of COVID-19 (coronavirus disease 2019) has now affected almost every region around the globe and continues to impact lives in all societies. Its causative pathogen, Severe Acute Respiratory Syndrome Coronavirus 2 (SARS-CoV-2), was first detected in China in late 2019, but has spread rapidly ever since causing significant burden of morbidity and mortality worldwide [1, 2]. Up until now, about six hundred million confirmed cases and over six million confirmed deaths were reported globally [3].

However, different countries presented different COVID-19 epidemics. Iran was one of the first countries hit by the virus, with the COVID-19 outbreak being reported initially in late February 2020. Like many other countries, massive public health measures have been imposed across the country since the beginning of the epidemic to contain the spread of the virus [4]. However, as of January 2022, the nation has experienced five consecutive waves of the disease in its epidemiological curve. The spread of the disease was successfully mitigated by implementing infection control policies in early stages of the epidemic. Yet, as the epidemic progressed, containing the virus did become more challenging and larger waves washed over the country. All in all, the Iranian epidemic had profound impact on its society with over seven million confirmed cases and about 140 thousand confirmed death tallies to date [5, 6].

Until now, incredible insight has been gained on COVID-19, but it has been mainly dominated by studies offering an overall view of the pandemic [7–10]. Yet, to better understand the evolving nature of the disease and its impacts on societies, a more detailed analysis of the pandemic’s characteristics is needed. Moreover, increasing our knowledge about policy responses which had successfully contained the spread of the virus could light our way in reducing future morbidity and mortality associated with the disease [11]. Hence, this study was conducted with the intension of providing a more detailed view about the dynamics of the COVID-19 pandemic. We aimed to assess the characteristics, implemented public health measures, and the health outcomes of COVID-19 patients during the five consecutive waves of the disease in Iran.

## Methods

This study was a population-based cross-sectional analysis of data on adult patients who were diagnosed with COVID-19 during the five waves of the disease in Tehran, Iran. With over 13 million residents, Tehran is the most populated province of Iran and an important epicentre for COVID-19 outbreaks in the country [12]. According to the Coronavirus Control Operations Headquarter in Tehran, the first wave hit the province on March 1, 2020, with daily average count of 904 newly infected patients and was ended on April 20, 2020, when the number of new daily cases fell to a low plateau. The start date for the second wave was recorded on July 4, 2020, as the new daily counts raised after remaining stable for over a month. An average of 725 cases per day were reported during the second wave and it ended on July 26, 2020. On September 19, 2020, a re-surge in new daily cases was detected, creating the huge third coronavirus wave in the province with an average daily case of 838. The third wave reached its plateau on November 25*,* 2020. The fourth wave began on March 31, 2021, during which an average of 929 cases were reported daily. The province managed to come out of it on May 29, 2021, however shortly after, on June 22 the country entered its fifth wave with the average daily count of 5825. The wave came to an end on December 18, 2021, after several weeks of declining numbers of new cases followed by a plateau.

The province-wide data on all patients aged 18 years or older infected with COVID-19 during the five consecutive waves of the epidemic were extracted from the registry database of Coronavirus Control Operations Headquarter in Tehran. Of note, as of late February 2020, all health care facilities visiting suspected, probable, and confirmed cases of COVID-19 patients (defined bases on WHO case definition guideline) in the province, had to use the COVID-19 online registry, where a standard online form with required and optional fields were directly filled during the visits by health care professionals [13]. In this study, a complete case analysis was performed by using data from variables with required fields including demographic characteristics, underlying diseases, clinical presentations, and the health outcomes of 1,254,747 adult patients. Hence, the rate of missing values and their effects were considered as insignificant.

The qualitative information including the implemented public health policies intended for virus containment were retrieved from the country’s official governmental sources (e.g. official website of Ministry of Health and Medical education in Iran, official website of medical education Universities responsible for conveying information about COVID-19) and a detailed catalogue of recommended and administered government policies related to COVID-19 which was available from the Coronavirus Control Operations Headquarter in Tehran.

### Variables

Variables used included age, sex, history of underlying diseases (diabetes, hypertension, cardiovascular disease, cancer, asthma, chronic liver disease, chronic kidney disease, chronic neurological disease, chronic haematological disease, chronic immune deficiency disease, history of smoking, history of opioid use), clinical presentations on admission (fever, cough, muscle ache, difficulty breathing, chest pain, loss of smell, loss of taste, loss of appetite, nausea, abdominal pain, diarrhea, headache, vertigo, seizure, paraplegia, skin lesions), blood oxygen saturation level (PaO_2_ sat %) on admission, polymerase chain reaction (PCR) test result on admission, chest CT findings on admission, intensive care unit (ICU) admission, health outcome, and the epidemiological waves.

The individuals were divided into 6 age groups: 18–24, 25–34, 35–44, 45–54, 55–64, and 65 or older. Sex was categorized as woman or man. The blood oxygen saturation level was recorded based on the National Coronavirus Treatment Guideline as being either higher than 93%, or 93% and lower. PCR test result was classified as negative, inconclusive, and positive. The outcome was defined as survived or deceased while in hospital. The epidemiological waves were coded from one to five. All other variables were documented as negative or positive.

### Statistical analysis

The characteristics of the study population were tabulated and presented using percentage for categorical variables and mean and standard deviation for continuous variables. Chi-squared test was used to compare the proportions of categorical variables between the five waves. One-way ANOVA was conducted to compare the continuous variables between the five waves. Logistic regression analysis was performed to adjust for the effect of possible confounding factors including age, sex, and underlying diseases on the health outcome. Statistical analyses were performed using IBM SPSS Statistics, version 27 (IBM Corp., Armonk, NY, USA), with significance level of α < 0.05.

### Ethics approval and consent to participate

Shahid Beheshti University of Medical Sciences Ethics Committee approved the study with a waiver of informed consent (Reference number: IR.SBMU.MSP.REC.1400.316). Data were de-identified prior to analysis. All methods were performed in accordance with the Declaration of Helsinki guidelines and regulations.

## Results

In total, data on 328,410 patients (46,271 from the first, 16,714 from the second, 57,146 from the third, 73,558 from the fourth, and 134,721 from the fifth wave) who were diagnosed with COVID-19 during five consecutive waves of the disease in Tehran were analysed. The mean age of patients was 53.5 ± 17.26 years (52.1 years for first, 55.4 years for second, 57.4 years for third, 55.7 years for fourth, and 50.9 for fifth wave). The one-way ANOVA revealed a significant difference in the mean age of patients between the groups (F_(4, 328405)_ = 1993.7, P < 0.001). Post hoc comparisons using the Scheffe test showed significant difference between mean age of patients among all comparisons (P < 0.001) except for difference between wave 2 and wave 4 which was insignificant (P = 0.23). Table 1 provides an overview of the COVID-19 epidemiological waves in Iran.

**Table 1:** COVID-19 epidemiological waves in Iran

	Wave 1	Wave 2	Wave 3	Wave 4	Wave 5
Start date (dd/mm/yyyy)	01/03/2020	04/06/2020	19/09/2020	31/03/2021	22/06/2021
End date (dd/mm/yyyy)	20/04/2020	26/06/2020	25/11/2020	29/05/2021	18/12/2021
Duration (days)	51	23	67	60	180
Daily average case count	904	725	838	929	5825
Mean age of patients (years)	52.1	55.4	57.4	55.7	50.9
Fatality average per day	89.1	95.0	134.3	96.5	29.4

At the beginning of the pandemic, there was a significant difference between men and women infected with COVID-19, with men having higher rates of the disease. But as the pandemic progressed, the differences between the sexes were lessened due to the gradual increase in the proportion of women who were infected with the virus during the first four waves. Subsequently during the fifth wave, more women were diagnosed with COVID-19 than men. Demographic characteristics of the study population is presented in Table 2.

**Table 2:** Demographic characteristics of the study population

Demographic characteristics	Wave 1	Wave 2	Wave 3	Wave 4	Wave 5	P value
	n, % (95% CI)	n, % (95% CI)	n, % (95% CI)	n, % (95% CI)	n, % (95% CI)	
Age (years)						
18–24	1919, 4.1 (3.2–4.9)	463, 2.8 (1.2–4.3)	1249, 2.2 (1.3–3.0)	1408, 1.9 (1.1–2.6)	4955, 3.7 (3.1–4.2)	< 0.001
25–34	6664, 14.4 (13.5–15.2)	1892, 11.3 (9.8–12.7)	5340, 9.3 (8.5–10.0)	6862, 9.3 (8.6–9.9)	18732, 13.9 (13.4–14.3)	
35–44	8994, 19.4 (18.5–20.2)	2747, 16.4 (15.0–17.7)	8348, 14.6 (13.8–15.3)	12969, 17.6 (16.9–18.2)	30518, 22.7 (22.2–23.1)	
45–54	8441, 18.2 (17.3–19.0)	2933, 17.5 (16.1–18.8)	9421, 16.5 (15.7–17.2)	13315, 18.1 (17.4–18.7)	26483, 19.7 (19.2–20.1)	
55–64	8038, 17.4 (16.5–18.2)	3113, 18.6 (17.2–19.9)	11282, 19.7 (18.9–20.4)	15208, 20.7 (20.0–21.3)	23799, 17.7 (17.2–18.1)	
65 +	12215, 26.4 (25.6–27.1)	5566, 33.3 (32.0–34.5)	21506, 37.6 (36.9–38.2)	23796, 32.3 (31.7–32.8)	30234, 22.4 (21.9–22.8)	
Sex						
Women	20654, 44.6 (43.9–45.2)	7605, 45.5 (44.3–46.6)	26152, 45.8 (45.1–46.4)	35352, 48.1 (47.5–48.6)	69866, 51.9 (51.5–52.2)	< 0.001
Men	25617, 55.4 (54.7–56.0)	9109, 54.5 (53.4–55.5)	30994, 54.2 (53.6–54.7)	38206, 51.9 (51.3–52.4)	64855, 48.1 (47.7–48.4)	
Total	46271, 100	16714, 100	57146, 100	73558, 100	134721, 100	

P value obtained from Chi squared test; %: Percentage in category; CI: Confidence Interval

In terms of underlying diseases, the prevalence of diabetes, hypertension, cardiovascular diseases, chronic neurological diseases, asthma, and having positive history for opioid use had increased during the first three waves, with the highest rates being reported in the third wave. Yet, the rates had steadily decreased during the next two successive waves. Greatest proportion of patients with positive history of smoking, cancers, and chronic haematological diseases were reported during the second wave. Comorbidities including chronic liver and chronic kidney diseases were equally prevalent in the waves two and three, but rates were significantly higher than that obtained during the other three waves (Table 3).

**Table 3:** Distribution of underlying diseases in patients with COVID-19 during the five consecutive waves of the disease

Underlying diseases	Wave 1	Wave 2	Wave 3	Wave 4	Wave 5	P value
	n, % (95% CI)	n, % (95% CI)	n, % (95% CI)	n, % (95% CI)	n, % (95% CI)	
Diabetes	3021, 6.5 (5.6–7.3)	1785, 10.7 (9.2–12.1)	7460, 13.1 (12.3–13.8)	8104, 11.0 (10.3–11.6)	10615, 7.9 (7.3–8.4)	** < **0.001
Hypertension	1476, 3.2 (2.3–4.0)	2033, 12.2 (10.7–13.6)	8666, 15.2 (14.4–15.9)	9820, 13.4 (12.7–14.0)	11298, 8.4 (7.8–8.9)	** < **0.001
Cardiovascular diseases	3146, 6.8 (5.9–7.6)	1591, 9.5 (8.0–10.9)	6259, 11.0 (10.2–11.7)	7286, 9.9 (9.2–10.5)	9142, 6.8 (6.2–7.3)	** < **0.001
Cancer	582, 1.3 (0.3–2.2)	326, 2.0 (0.4–3.5)	1060, 1.9 (1.0–2.7)	1005, 1.4 (0.6–2.1)	1397, 1.0 (0.4–1.5)	** < **0.001
Asthma	518, 1.1 (0.2–1.9)	207, 1.2 (0.2–2.6)	786, 1.4 (0.5–2.2)	771, 1.0 (0.2–1.7)	875, 0.6 (0.0–1.1)	** < **0.001
Chronic liver diseases	164, 0.4 (0.0–1.3)	81, 0.5 (0.0–2.0)	291, 0.5 (0.0–1.3)	307, 0.4 (0.0–1.1)	489, 0.4 (0.0–0.9)	** < **0.001
Chronic kidney diseases	642, 1.4 (0.4–2.3)	352, 2.1 (0.6–3.5)	1194, 2.1 (1.2–2.9)	974, 1.3 (0.5–2.0)	1401, 1.0 (0.4–1.5)	** < **0.001
Chronic neurological diseases	304, 0.7 (0.0 1.6)	147, 0.9 (0.0–2.4)	581, 1.0 (0.1- 1.8)	454, 0.6 (0.0- 1.3)	614, 0.5 (0.0- 1.0)	** < **0.001
Chronic immune deficiency diseases	125, 0.3 (0.0–1.2)	57, 0.3 (0.0–1.7)	141, 0.2 (0.0–0.9)	201, 0.3 (0.0–1.0)	229, 0.2 (0.0- 0.7)	** < **0.001
Chronic hematological diseases	160, 0.3 (0.0- 1.1)	85, 0.5 (0.0–1.9)	253, 0.4 (0.0–1.1)	226, 0.3 (0.0–1.0)	413, 0.3 (0.0–0.8)	** < **0.001
Positive history of smoking	456, 1.0 (0.0–1.9)	307, 1.8 (0.3–3.2)	927, 1.6 (0.7–2.4)	1091, 1.5 (0.7–2.2)	1786, 1.3 (0.7–1.8)	** < **0.001
Positive history of opioids	223, 0.5 (0.0–1.4)	129, 0.8 (0.0–2.3)	523, 0.9 (0.0–1.7)	482, 0.7 (0.0–1.4)	818, 0.6 (0.0–1.1)	** < **0.001

P value obtained from Chi squared test; %: Percentage in category; CI: Confidence Interval

The distribution of patients’ physical complaints throughout the five waves of the epidemic is shown in Table 4. The most frequently recorded clinical presentation during the first and second wave were cough, difficulty breathing, and fever. However, the three most common symptoms during the third and fourth wave were cough, difficulty breathing, and muscle ache. Fever, abdominal pain, and diarrhea were most prevalent in the second wave. Complaints of difficulty breathing, muscle ache, loss of smell, loss of taste, and nausea were highest during the third wave. Highest rates for cough, headache, vertigo, and loss of appetite were observed during the fourth wave. Overall, conditions including seizure, paraplegia, and skin lesions were not commonly reported during the epidemic.

**Table 4:** Clinical presentation in patients with COVID-19 during the five consecutive waves of the disease

Clinical findings	Wave 1	Wave 2	Wave 3	Wave 4	Wave 5	P value
	n, % (95% CI)	n, % (95% CI)	n, % (95% CI)	n, % (95% CI)	n, % (95% CI)	
Fever	15484, 33.5 (32.7–34.2)	6361, 38.1 (36.9–39.2)	19302, 33.8 (33.1–34.3)	25139, 34.2 (33.6–34.7)	44329, 32.9 (32.4–33.3)	** < **0.001
Cough	22252, 48.1 (47.4–48.7)	7606, 45.5 (44.3–46.6)	28933, 50.6 (50.0–51.1)	40894, 55.6 (55.1–56.0)	81404, 60.4 (60.0–60.7)	** < **0.001
Muscle ache	11214, 24.2 (23.4–24.9)	5633, 33.7 (32.4–34.9)	21470, 37.6 (36.9–38.2)	27287, 37.1 (36.5–37.6)	59508, 44.2 (43.8–44.5)	** < **0.001
Difficulty breathing	16600, 35.9 (35.1–36.6)	7579, 45.3 (44.1–46.4)	29419, 51.5 (50.9–52.7)	34871, 47.4 (46.8–47.9)	44290, 32.9 (32.4–33.3)	** < **0.001
Chest pain	31, 0.1 (0.0–4.5)	536, 3.2 (1.7–4.6)	2339, 4.1 (3.2–4.9)	2656, 3.6 (2.8–4.3)	3521, 2.6 (2.0–3.1)	** < **0.001
Loss of smell	423, 0.9 (0.0–1.8)	449, 2.7 (1.2–4.1)	1738, 3.0 (2.1–3.8)	1915, 2.6 (1.8–3.3)	4693, 3.5 (2.9–4.0)	** < **0.001
Loss of taste	172, 0.4 (0.0–1.3)	261, 1.6 (0.0–3.1)	1210, 2.1 (1.2–2.9)	964, 1.3 (0.5–2.0)	3144, 2.3 (1.7–2.8)	** < **0.001
Loss of appetite	64, 0.1 (0.0–0.8)	1127, 6.7 (5.2–8.1)	4602, 8.1 (7.3–8.8)	7101, 9.7 (9.0–10.3)	13451, 10.1 (9.5–10.6)	** < **0.001
Nausea	74, 0.2 (0.0–1.2)	1079, 6.5 (5.0–7.9)	4220, 7.4 (6.6–8.1)	4940, 6.7 (6.0–7.3)	9989, 7.4 (6.8–7.9)	** < **0.001
Abdominal pain	26, 0.1 (0.0–1.3)	487, 2.9 (1.4–4.3)	1563, 2.7 (1.8–3.5)	1981, 2.7 (1.9–3.4)	3214, 2.4 (1.8–2.9)	** < **0.001
Diarrhea	32, 0.1 (0.0–1.1)	798, 4.8 (3.3–6.2)	1932, 3.4 (2.5–4.2)	2132, 2.9 (2.1–3.6)	2846, 2.1 (1.5–2.6)	** < **0.001
Headache	96, 0.2 (0.0–1.0)	1170, 7.0 (5.5–8.4)	5514, 9.6 (8.8–10.3)	7898, 10.7 (10.0–11.3)	17521, 13.0 (12.5–13.4)	** < **0.001
Vertigo	20, 0.0 (0.0–0.0)	456, 2.7 (1.2–4.1)	1789, 3.1 (2.2–3.9)	2758, 3.7 (2.9–4.4)	4834, 3.6 (3.0–4.1)	** < **0.001
Seizure	27, 0.1 (0.0–1.2)	42, 0.3 (0.0–1.9)	115, 0.2 (0.0–1.0)	97, 0.1 (0.0–0.7)	180, 0.1 (0.0–0.7)	** < **0.001
Paraplegia	5, 0.0 (0.0–0.0)	23, 0.1 (0.0–1.3)	108, 0.2 (0.0–1.0)	96, 0.1 (0.0–0.7)	138, 0.1 (0.0–0.6)	** < **0.001
Skin lesions	1, 0.0 (0.0–0.0)	17, 0.1 (0.0–1.6)	60, 0.1 (0.0–0.8)	50, 0.1 (0.0–0.9)	121, 0.1 (0.0–0.6)	** < **0.001

P value obtained from Chi squared test; %: Percentage in category; CI: Confidence Interval

Regarding para-clinical findings in patients infected with COVID-19, a decreasing trend was observed in the proportion of patients experiencing low levels of blood oxygen saturation up until the fifth wave where a dramatic increase was observed in the proportion of patients presenting with low levels of PaO2 saturation. However, rates for positive PCR test results and COVID-19 related findings in chest CT has increased as the epidemic has unfolded. With respect to the health outcome of patients diagnosed with COVID-19, the rates of ICU admission and death have gradually increased during the first three waves but has significantly decreased during the last two waves (Table 5).

**Table 5:** Para-clinical findings and the health outcome of patients diagnosed with COVID-19 during the five consecutive waves of the disease

Para-clinical findings	Wave 1	Wave 2	Wave 3	Wave 4	Wave 5	P value
	n, % (95% CI)	n, % (95% CI)	n, % (95% CI)	n, % (95% CI)	n, % (95% CI)	
PaO_2_ sat < 93	27445, 59.3 (58.7–59.8)	7970, 47.7 (46.4–48.7)	24867, 43.5 (42.8–44.1)	29695, 40.4 (39.8–40.9)	109105, 81.0 (80.7–81.2)	** < **0.001
PCR test result						
Positive	11000, 23.8 (23.0–24.5)	6592, 39.4 (38.2–40.5)	25743, 45.0 (44.3–45.6)	39500, 53.7 (53.2–54.1)	62236, 49.4 (49.0–49.7)	** < **0.001
Inconclusive	23112, 49.9 (49.2–50.5)	5112, 30.6 (29.3–31.8)	17126, 30.0 (29.3–30.6)	23256, 31.6 (31.0–32.1)	45071, 35.7 (35.2–36.1)	
Negative	12159, 26.3 (25.5–27.0)	5010, 30.0 (28.7–31.2)	14277, 25.0 (24.2–25.7)	10802, 14.7 (14.0–15.3)	18793, 14.9 (14.3–15.4)	
Positive findings in Chest CT	13937, 30.1 (29.3–30.8)	10958, 65.6 (64.7–66.4)	40210, 70.4 (69.9–70.8)	56450, 76.7 (76.3–77.0)	99886, 74.1 (73.8–74.3)	** < **0.001
ICU admission	6773, 14.7 (13.8–15.5)	2943, 17.6 (16.2–18.9)	10763, 18.8 (18.0–19.5)	10786, 14.7 (14.0–15.3)	13578, 10.1 (9.5–10.6)	** < **0.001
Death	4546, 9.8 (8.9–10.6)	2187, 13.1 (11.6–14.5)	9000, 15.7 (14.9–16.4)	5793, 7.9 (7.2–8.5)	5305, 3.9 (3.3–4.4)	** < **0.001

P value obtained from Chi squared test; %: Percentage in category; CI: Confidence Interval

After accounting for age, sex, and underlying diseases in the logistic regression analysis, the probability of dying with COVID-19 has increased during the first three waves, with highest odds ratio observed during the third wave after which the likelihood of dying as the result of the infection has considerably declined during the next two waves (Table 6).

**Table 6:** Logistic regression model of independent variables associated with COVID-19 mortality

Variables	aOR	95% confidence interval		P value
		Lower	Upper	
Age (years)				
18–24	1			
25–34	0.91	0.758	1.106	0.36
35–44	1.61	1.353	1.922	< 0.001
45–54	3.03	2.556	3.605	< 0.001
55–64	5.44	4.594	6.455	< 0.001
65 +	13.09	11.065	15.497	< 0.001
Sex				
Women	1			
Men	1.29	1.263	1.332	< 0.001
Positive history of smoking	0.81	0.727	0.914	< 0.001
Positive history of opioids	1.25	1.095	1.442	< 0.001
Underlying disease				
Diabetes	1.15	1.110	1.200	< 0.001
Hypertension	1.07	1.033	1.114	< 0.001
Cardiovascular diseases	1.15	1.113	1.203	< 0.001
Cancer	2.27	2.100	2.457	< 0.001
Asthma	0.89	0.792	1.010	0.72
Chronic liver diseases	1.81	1.549	2.116	< 0.001
Chronic kidney diseases	2.08	1.929	2.243	< 0.001
Chronic neurological diseases	1.74	1.553	1.963	< 0.001
Chronic immune deficiency diseases	1.38	1.087	1.766	0.003
Chronic hematological diseases	1.41	1.182	1.684	< 0.001
Waves				
Wave 1	1			
Wave 2	1.16	1.104	1.238	< 0.001
Wave 3	1.34	1.287	1.395	< 0.001
Wave 4	0.648	0.621	0.677	< 0.001
Wave 5	0.389	0.372	0.406	< 0.001
Constant	0.01			< 0.001

**Fig. 1:**
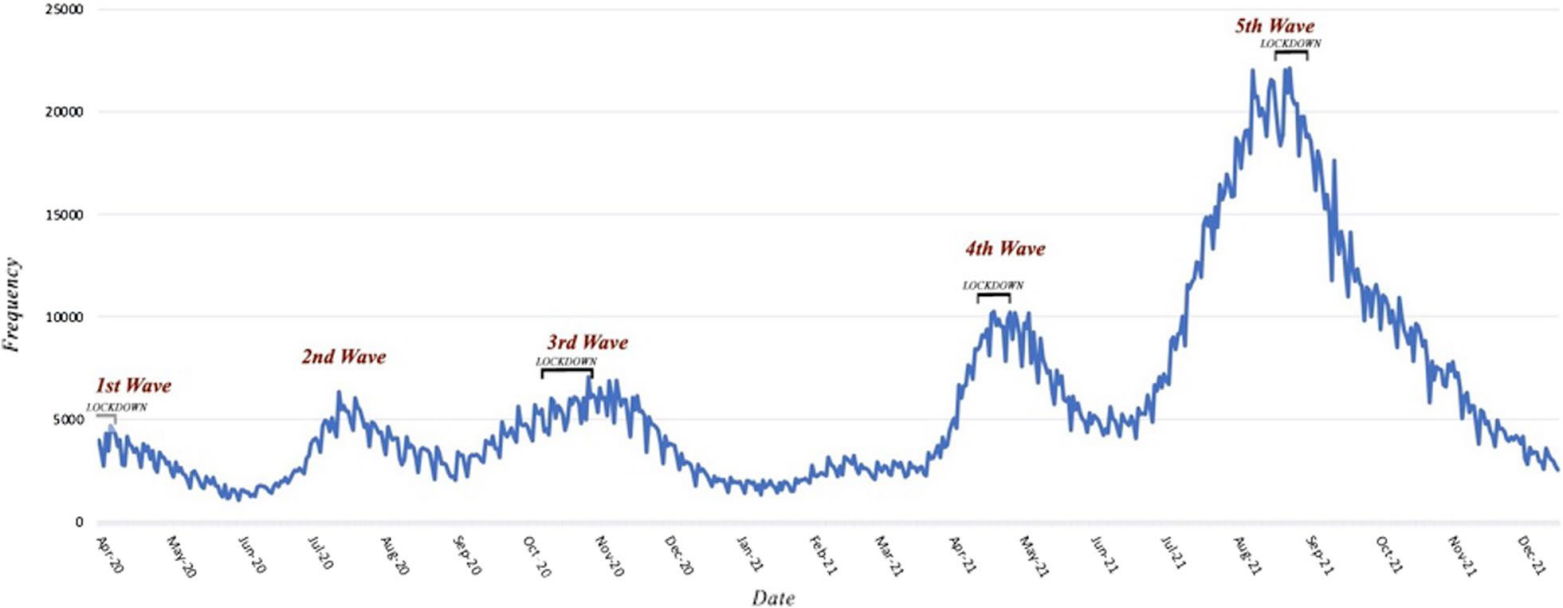
Frequency of patients diagnosed with COVID-19 in the province of Tehran, Iran from March 2020 to December 2021

With respect to COVID-19 coping strategies implemented by the government during the epidemic, the first public health approach was a complete lockdown which took place on March 5, 2020, only few days after the province had officially entered in to its first wave. The complete lockdown was defined as closure of all non-essential services, all public and private organizations, businesses, and recreational facilities. The complete lockdown was in place until April 3, 2020, and was accompanied by a good compliance from public side. As of April 3rd, businesses started operating and few weeks later travel restrictions and capacity limits were lifted. Universities and schools reopened and compliance with infection control policies including mask mandates and social distancing decreased to as low as 51% of the total population. All these contributed to the resurgence of new daily cases and initiation of the second wave. However, complete closures never happened during the second wave. The virus spread was contained by adopting a combination of public health measures including effective communication strategies with the goal of raising public awareness about COVID-19 containment measures, massive screening policies, quarantine and temporary closure of facilities reporting outbreaks. Nonetheless it worth mentioning that all schools and most universities across the province were closed by the time the second wave had reached its peak due to the provincial summer break. Even after the summer break, on September 5, 2020, when the schools and universities re-opened, the needed infrastructure for online education was well functional across the province with only 15 per cent of students attending in person classes. Yet, the third wave prompted by holding religious mass gatherings in the province in late August and was hardly controlled by soft public health measures including mask mandates, physical distancing, and social gathering limitations. Eventually, despite resistance in different governmental sectors a strict lockdown went into effect on October 4, 2020, lasting for 20 days. After which all public and private organizations, businesses, and recreational facilities were allowed to operate with 50 percent capacity and were obligated to close by 6:00 pm. This epidemic control policy could effectively limit the spread of the virus causing case counts to sink again. However, the province entered a fourth wave shortly after the Iranian new year holidays in late March 2021, which was mainly linked with increased family gatherings and domestic travels. The rapidly rising spread of the virus led to the enforcement of another complete lockdown in the province effective from April 10, 2021, to April 24, 2021. The containment policy included inter-city travel restriction (limiting travelling between cities to essential purposes only) as well as social gathering limits of 15 person indoor [14]. However, just as the numbers started to sink, the low adherence to public health measures and concurrence invasion of a new variant of the virus caused case counts climb for the fifth time in a row on June 22, 2021 [15]. In order to control this huge wave, all non-essential businesses were closed for six days starting from July 20, 2021, which was considered by many as ineffective. Yet, the unprecedented size of the resurgence led to implementation of the fourth complete lockdown on August 15 including inter- and intra-city mobility restrictions which was in effect for two weeks. Intra-city restriction barred overnight mobility within the cities from 10:00 pm to 3:00 am with a few exemptions and was in place for a couple of months after the lockdown has ended [16]. Ultimately, the province’s biggest wave of COVID-19 relented months after the province-wide restrictions were first imposed (Fig. 1).

## Discussion

This study is a population-based cross-sectional analysis of data on 328,410 adult patients who were diagnosed with COVID-19 during five consecutive waves of the disease in Tehran. Main findings indicated that the probability of dying with COVID-19 has increased as the pandemic wore on, showing its highest odd during the third wave and has gradually decreased during the next two waves. The same pattern was observed in the proportion of patients requiring ICU admission. Overall, the first wave presented mainly with respiratory symptoms, and gastrointestinal complaints were added during the second wave. During the third wave, neurological manifestations with peripheral involvement replaced the gastrointestinal complaints, and central nervous system manifestations were added during the next two waves. With respect to containment public health policies, adopting complete or targeted lockdowns were the key measures that helped to mitigate the virus spread in Iran.

Our findings revealed that more severe cases have been reported during the third wave, compared to the other waves. After adjusting for age, sex, and underlying diseases in the logistic regression analysis, the odds of dying with COVID-19 has increased during the first three waves but has significantly decreased during the next two waves. The same pattern was seen in South Korea, Australia, and France which were experiencing their second wave of COVID-19 during the same period when Iran was in its third wave, suggesting that as of early August, a more virulent and fatal variant of the virus was circulating in most countries around the globe [17–19]. A recent reduction in COVID-19 related mortality rate has also been documented in studies evaluating the trends in death rate due to COVID-19 [20–22]. Generally, it is well established that viruses including SARS-CoV-2 naturally mutate over time affecting their transmissibility and virulence [23]. According to experts’ report, in Iran the first wave was caused by the wild variant imported from China, the second wave was mainly due to a mutated variant which has travelled from China to western states and then has entered the country, namely B.1.36. The third wave was mainly related to a variant of concern, B.1.1.413 first identified in western countries including Europe and Canada. Alpha variant first identified in England was the dominant variant in the fourth wave and the fifth wave was mainly caused by Delta variant first detected in India [24]. Our findings indicated that the fatality rate had significantly decreased by emergence of Alpha variant and consequently the Delta variant. Reduction in fatality rate over time could also be attributed to the increased protection against the disease provided by previous immunity, be it infection, vaccination, or a combination of both. As per Statistic Center of Iran, the national vaccination rollout plan began before the arrival of the fourth wave. Though it had minor effect on controlling high case counts during this period due to slow vaccination pace, it could significantly decrease death rates observed during the fourth and fifth wave of the disease by prioritizing high-risk populations including seniors, health-care workers working in close contact with COVID-19 patients, and individuals with chronic diseases in early phases of its rollout plan. The vaccination coverage of eligible population, i.e., two doses in individuals eighteen years and above, was only about 5% by the time the fifth wave began and a coverage of about 65 percent of eligible population was reached in December 2021 by the time the fifth wave reached its low plateau. Yet, in terms of the proportion of COVID-recovered individuals in different time periods in Iran, no accurate information has been made available so far [25].

Moreover, this study found changes in the patterns of COVID-19 symptoms as the epidemic evolved in the country. Overall, few studies have evaluated the changes in the COVID-19 presentation during the different pandemic waves, but the overall changing pattern of the symptoms have been previously reported [26, 27]. This could again be related to the ongoing evolution of SARA-CoV-2 as it circulated among populations [28]. Clinical manifestations included mainly respiratory symptoms when COVID-19 first appeared in the country, however, gastrointestinal complaints were added during the second wave. This was in line with a previous study conducted earlier in the country [29]. During the third wave, neurological manifestations with peripheral involvement replaced the gastrointestinal complaints, and central nervous system manifestations were added during the fourth and fifth waves. Overall, cough, difficulty breathing, and fever remained the leading complaints throughout the pandemic. However, the prevalence of muscle ache increased gradually since the first wave and replaced fever as the pandemic unfolded, indicating the inclusion of less severe cases in the COVID-19 surveillance system. At the beginning of the pandemic limited COVID-19 test kits were available, therefore like many other countries with inadequate testing capacities, testing was restricted to symptomatic patients. Yet, as the epidemic wore on, more PCR test kits became available allowing the evaluation of those with mild or no symptoms [30].

Our results revealed a demographic shift towards more younger people and more women being affected by the illness as the epidemic progressed, which was aligned with reports on changing demographics of the pandemic from other countries [31–34]. The shift in the age category of patients could be accounted for in part by national vaccination program as it was focused initially on the elderlies. However, the reasons behind the rising proportion of women diagnosed with COVID-19 are not yet entirely understood. One possible explanation could be the higher ratio of women who work at frontline industries including health care, childcare, social services, cleaning services, and administrative roles which might put them at increased risk of contracting the virus [35]. As the pandemic continued, more people including women had to return to work, leading to the rising trend in proportion of women infected with the virus observed in this study. However, to further clarify this issue future studies are recommended.

Iran is among the few countries with five waves of the disease in its COVID-19 epidemiological curve causing the government to take a wide range of public health measures in their response to outbreaks. Throughout the pandemic, a descending trend in new case counts was observed each time a complete lockdown was adopted. This observation points to the probability that complete lockdowns were the key measures that helped to mitigate the virus spread and to initiate the descending phase of the epidemiological curve. Though our finding lends support to previous reports in the literature, the association should be interpreted with caution since it could be affected by different interfering factors [36–38]. Overall, despite successfully implementing restrictive containment mandates at the beginning, the health sector faced increasing governance challenges as the pandemic proceeded. The major challenge was the growing resistance in different sectors in implementing proposed containment strategies out of great concerns about their socio-economic consequences. Overtime increase in public non-compliance with infection control policies has contributed to prolonged waves and shortened plateaus in the country’s COVID-19 epidemiological curve.

Finally, our research had few potential limitations. First, this study was a retrospective analysis of existing data which has restricted our analyses to available information. Future studies evaluating the effects of respiratory supports and other approved therapies on the health outcome of SARS-CoV-2 infected patients are recommended. Second, COVID-19 waves have occurred in different periods, hence there could be different confounding factors which could influence the health outcome of patients in different stages including use of therapies associated with improved health outcomes over time, proportion of vaccinated or previously infected individuals, changes in population behaviours, exhaustion of healthcare workforce, and strains on health system resources containing ICU or total bed capacity. Third, since the government had rolled out several public health interventions simultaneously during the peaks, detangling the impact of each intervention was challenging. And fourth, given the large sample size even small differences might become detectable, however, that statistical significance may not always imply something practically meaningful. Despite these drawbacks, by using a large provincial representative study population and a high-quality data set, collected by trained health care personnel based on a standardized reporting form, we were able to provide a reliable picture of the COVID-19 epidemic in the country allowing more confident extension of inferences to the target population. Moreover, consulting with authorities directly involved in COVID-19 related public policy making, enabled us to provide a more in-depth review of the implemented strategies.

## Conclusion

This study revealed a changing pattern in clinical manifestations and severity of COVID-19 pandemic during the five consecutive waves of the disease in Iran during 2020 and 2021. COVID-19 related public health interventions have also been reviewed, indicating the importance of population-wide COVID-19 immunization coverage and early implementation of infection control measures as effective strategies of virus containment.

## Data Availability

The data underlying this article were provided by Coronavirus Control Operations Headquarter in the province of Tehran under licence. Data will be shared on request to the corresponding author with permission of Coronavirus Control Operations Headquarter in the province of Tehran.

## Abbreviations

COVID-19: Coronavirus disease 2019
SARS-CoV-2: Severe acute respiratory syndrome coronavirus 2
PaO2 sat %: Blood oxygen saturation level
PCR: Polymerase chain reaction
ICU: Intensive care unit

## References

[1] World Health Organization. Naming the coronavirus disease (COVID-19) and the virus that causes it 2021. Available from: https://www.who.int/emergencies/diseases/novel-coronavirus-2019/technical-guidance/naming-the-coronavirus-disease-(covid-2019)-and-the-virus-that-causes-it.

[2] World Health Organization. Timeline: WHO's COVID-19 response 2021. Available from: https://www.who.int/emergencies/diseases/novel-coronavirus-2019/interactive-timeline.

[3] World Health Organization. Coronavirus disease (COVID-19) pandemic 2021. Available from: https://www.who.int/emergencies/diseases/novel-coronavirus-2019. 37184163

[4] Takian A, Raoofi A, Kazempour-Ardebili S (2020). COVID-19 battle during the toughest sanctions against Iran. Lancet.

[5] World Health Organization. Iran (Islamic Republic of) 2021. Available from: https://www.who.int/countries/irn/.

[6] Devi S (2020). COVID-19 resurgence in Iran. Lancet.

[7] Fang X, Li S, Yu H, Wang P, Zhang Y, Chen Z (2020). Epidemiological, comorbidity factors with severity and prognosis of COVID-19: a systematic review and meta-analysis. Aging (Albany NY).

[8] Nikpouraghdam M, Jalali Farahani A, Alishiri G, Heydari S, Ebrahimnia M, Samadinia H (2020). Epidemiological characteristics of coronavirus disease 2019 (COVID-19) patients in IRAN: a single center study. J Clin Virol.

[9] Kumar A, Arora A, Sharma P, Anikhindi SA, Bansal N, Singla V (2020). Clinical features of COVID-19 and factors associated with severe clinical course: a systematic review and meta-analysis. SSRN.

[10] Abate SM, Ahmed Ali S, Mantfardo B, Basu B (2020). Rate of intensive care unit admission and outcomes among patients with coronavirus: a systematic review and meta-analysis. PLoS ONE.

[11] Van Damme W, Dahake R, Delamou A, Ingelbeen B, Wouters E, Vanham G (2020). The COVID-19 pandemic: diverse contexts; different epidemics-how and why?. BMJ Glob Health.

[12] Statistical Center of Iran. Population and Housing Censuses 2021. Available from: https://www.amar.org.ir/english/Population-and-Housing-Censuses.

[13] World Health Organization. WHO COVID-19 Case definition 2020 [Available from: https://www.who.int/publications/i/item/WHO-2019-nCoV-Surveillance_Case_Definition-2020.1.

[14] Donya-e-eqtesad. Announcement of corona restrictions in the fourth corona peak + photo 2021. Available from: https://donya-e-eqtesad.com/%D8%A8%D8%AE%D8%B4-%D8%B3%D8%A7%DB%8C%D8%AA-%D8%AE%D9%88%D8%A7%D9%86-62/3754410-%D8%A7%D8%B9%D9%84%D8%A7%D9%85-%D9%85%D8%AD%D8%AF%D9%88%D8%AF%DB%8C%D8%AA-%D9%87%D8%A7%DB%8C-%DA%A9%D8%B1%D9%88%D9%86%D8%A7%DB%8C%DB%8C-%D8%AF%D8%B1-%D9%BE%DB%8C%DA%A9-%DA%86%D9%87%D8%A7%D8%B1%D9%85-%DA%A9%D8%B1%D9%88%D9%86%D8%A7-%D8%B9%DA%A9%D8%B3.

[15] Khabaronline. When will the fourth wave of the corona descend?/Announce the decision time to continue the corona restrictions 2021. Available from: https://www.khabaronline.ir/news/1506019/%D9%85%D9%88%D8%AC-%DA%86%D9%87%D8%A7%D8%B1%D9%85-%DA%A9%D8%B1%D9%88%D9%86%D8%A7-%DA%A9%DB%8C-%D9%86%D8%B2%D9%88%D9%84%DB%8C-%D9%85%DB%8C-%D8%B4%D9%88%D8%AF-%D8%A7%D8%B9%D9%84%D8%A7%D9%85-%D8%B2%D9%85%D8%A7%D9%86-%D8%AA%D8%B5%D9%85%DB%8C%D9%85-%DA%AF%DB%8C%D8%B1%DB%8C-%D8%A8%D8%B1%D8%A7%DB%8C.

[16] donya-e-eqtesad. Tehran on the fifth day of coronary restrictions. 2021.

[17] Seong H, Hyun HJ, Yun JG, Noh JY, Cheong HJ, Kim WJ (2021). Comparison of the second and third waves of the COVID-19 pandemic in South Korea: importance of early public health intervention. Int J Infect Dis.

[18] Jarrett SA, Lo KB, Shah S, Zanoria MA, Valiani D, Balogun OO (2021). Comparison of patient clinical characteristics and outcomes between different COVID-19 peak periods: a single center retrospective propensity matched analysis. Cureus.

[19] World Health Organization. France Situation 2021. Available from: https://covid19.who.int/region/euro/country/fr.

[20] Madahar P, Wunsch H, Jha P, Slutsky AS, Brodie D (2021). Trends in COVID-19-related in-hospital mortality: lessons learned from nationwide samples. Lancet Respir Med.

[21] Horwitz LI, Jones SA, Cerfolio RJ, Francois F, Greco J, Rudy B (2021). Trends in COVID-19 risk-adjusted mortality rates. J Hosp Med.

[22] James N, Menzies M (2021). Trends in COVID-19 prevalence and mortality: a year in review. Physica D.

[23] Zawbaa HM, Osama H, El-Gendy A, Saeed H, Harb HS, Madney YM (2022). Effect of mutation and vaccination on spread, severity, and mortality of COVID-19 disease. J Med Virol.

[24] Agency IRN. Which aspects of the corona spread in Iran? 2021 [Available from: https://www.irna.ir/news/84571436/%DA%A9%D8%AF%D8%A7%D9%85-%D8%B3%D9%88%DB%8C%D9%87-%D9%87%D8%A7%DB%8C-%DA%A9%D8%B1%D9%88%D9%86%D8%A7-%D8%AF%D8%B1-%D8%A7%DB%8C%D8%B1%D8%A7%D9%86-%D8%B4%DB%8C%D9%88%D8%B9-%D9%BE%DB%8C%D8%AF%D8%A7-%DA%A9%D8%B1%D8%AF%D9%86%D8%AF.

[25] Presidency of the I.R.I Plam and Budget Organization. Statistic Center of Iran 2021. Available from: https://www.amar.org.ir/english.

[26] Firouzabadi FD, Firouzabadi MD, Ghalehbaghi B, Jahandideh H, Roomiani M, Goudarzi S (2020). Have the symptoms of patients with COVID-19 changed over time during hospitalization?. Med Hypotheses.

[27] Del Rio C, Malani PN (2020). COVID-19-new insights on a rapidly changing epidemic. JAMA.

[28] Lauring AS, Hodcroft EB (2021). Genetic variants of SARS-CoV-2-what do they mean?. JAMA.

[29] Sohrabi MR, Amin R, Maher A, Bahadorimonfared A, Janbazi S, Hannani K (2021). Sociodemographic determinants and clinical risk factors associated with COVID-19 severity: a cross-sectional analysis of over 200,000 patients in Tehran. Iran BMC Infect Dis.

[30] Murphy A, Abdi Z, Harirchi I, McKee M, Ahmadnezhad E (2020). Economic sanctions and Iran's capacity to respond to COVID-19. Lancet Public Health.

[31] Taylor L (2021). COVID-19: Brazil's spiralling crisis is increasingly affecting young people. BMJ.

[32] Government of Canada. COVID-19 daily epidemiology update 2022. Available from: https://health-infobase.canada.ca/covid-19/epidemiological-summary-covid-19-cases.html#a5.

[33] Bird PW, Riff R, Folwell A, Holmes CW, Tang JW (2021). Increased incidence of COVID-19 in younger patients (May–July 2021)—an argument for extending vaccination?. J Med Virol..

[34] Goldstein E, Lipsitch M (2020). Temporal rise in the proportion of younger adults and older adolescents among coronavirus disease (COVID-19) cases following the introduction of physical distancing measures, Germany, March to April 2020. Euro Surveill..

[35] Hye J, Rho HB, Fremstad S. A Basic Demographic Profile of Workers in Frontline Industries: Center for Economic and Policy Research; 2020. Available from: https://cepr.net/wp-content/uploads/2020/04/2020-04-Frontline-Workers.pdf.

[36] Rassouli M, Ashrafizadeh H, Shirinabadi Farahani A, Akbari ME (2020). COVID-19 management in Iran as one of the most affected countries in the world: advantages and weaknesses. Front Public Health.

[37] Ansah JP, Matchar DB, Shao Wei SL, Low JG, Pourghaderi AR, Siddiqui FJ (2021). The effectiveness of public health interventions against COVID-19: lessons from the Singapore experience. PLoS ONE.

[38] Hartley DM, Perencevich EN (2020). Public health interventions for COVID-19: emerging evidence and implications for an evolving public health crisis. JAMA.

